# Nanoporous Cyanate Ester Resins: Structure-Gas Transport Property Relationships

**DOI:** 10.1186/s11671-017-2071-3

**Published:** 2017-04-26

**Authors:** Kristina Gusakova, Alexander Fainleib, Eliane Espuche, Olga Grigoryeva, Olga Starostenko, Fabrice Gouanve, Gisèle Boiteux, Jean-Marc Saiter, Daniel Grande

**Affiliations:** 1 Institute of Macromolecular Chemistry, National Academy of Sciences of Ukraine, 48 Kharkivske shose, Kyiv, 02160 Ukraine; 2 “Ingénierie des Matériaux Polymères”, UMR 5223 CNRS-UCBL-INSA Lyon-UJM, Université de Lyon, 69622 Villeurbanne, France; 3 Université de Rouen, SMS UPRES EA 3233, IRCOF, 1 rue Tesnière, 76821 Mont Saint Aignan, France; 4 Institut de Chimie et des Matériaux Paris-Est, UMR 7182 CNRS, Université Paris-Est Créteil Val-de-Marne, 94320 Thiais, France

**Keywords:** Cyanate ester resins, Nanoporous thermosets, Gas solubility, Gas diffusion, Transport properties

## Abstract

This contribution addresses the relationships between the structure and gas transport properties of nanoporous thermostable cyanate ester resins (CERs) derived from polycyclotrimerization of 1,1′-bis(4-cyanatophenyl)ethane in the presence of 30 or 50 wt% of inert high-boiling temperature porogens (i.e., dimethyl- or dibutyl phthalates), followed by their quantitative removal. The nanopores in the films obtained were generated via a chemically induced phase separation route with further porogen extraction from the densely crosslinked CERs. To ensure a total desorption of the porogen moieties from the networks, an additional short-term thermal annealing at 250 °C was performed. The structure and morphology of such nanoporous CER-based films were investigated by FTIR and SEM techniques, respectively. Further, the gas transport properties of CER films were analyzed after the different processing steps, and relationships between the material structure and the main gas transport parameters were established.

## Background

Nowadays, cyanate ester resins (CERs) become more and more well known because they display a unique combination of high-performance characteristics, such as excellent thermal and chemical stability, durability at elevated temperatures, superior adhesion to various substrates, and low dielectric losses, in comparison with that of conventional thermosets [1–3]. Therefore, CERs and CER-based nanomaterials are widely used for producing high-temperature structural and functional units in aerospace and microelectronics, e.g., as composite matrices for strakes, fins, nose radomes, heat shields, printed circuit boards, and as encapsulants and adhesives [4]. One could also expect a great potential of porous CER materials as thermostable membranes, filters, sensors, or sorbents in filtration, separation, and purification processes requiring severe conditions. However, to the best of our knowledge, CER materials have not been applied as (nano)porous media for membrane technologies so far. Contrarily, thermostable porous polyimides, for example, are well-known multipurpose membranes in different branches of industry [5–7].

Pioneering studies on the generation of porous CER-based materials were published by Hedrick et al. and Kiefer et al. in 1996 [8–11]. They actually applied the chemically and thermally induced phase separation approaches to the production of micro- and macroporous CERs. Hedrick et al. [8] developed porous CER-containing samples via polycyclotrimerization of 4,4′-(hexafluoroisopropylidene) diphenyl-cyanate *in situ* with reactive poly(propylene oxide) (PPO) or PPO-based polyurethane as thermally labile porogens. During the CER network synthesis, the aforementioned modifiers used were fully incorporated into the CER structure, resulting in the formation of hybrid CER networks. However, the authors found that the structural order was broken following porogen thermolysis, due to a significant increase in the mobility of CER fragments while PPO degradation took place, thus resulting in partial pore collapse [8].

On the other hand, Kiefer et al. described the synthesis of CER foams using cyclohexane as a porogen [9, 10]. Foam creation as well as full network curing were accomplished by chemically induced phase separation realized through heating of cyanate ester/cyclohexane mixture up to 280 °C in vacuum. The SEM micrographs confirmed the presence of interconnected porous structure with round pores having diameter from 10 to 20 μm. Later on, several works [12, 13] on the influence of porogen type and amount, synthesis conditions and phase separation technique on chemical structure, morphological peculiarities, and basic physical chemical properties (thermal stability, dielectric constant, etc.) of porous CERs produced were published as well. In these investigations, either membrane or barrier and adsorption properties were not studied, possibly due to complexity of obtaining CER-based thin film materials.

Only in the past few years, several attempts to generate triazine-based microporous polymer materials for gas sorption processes were realized [14–18]. A microporous structure with major pore size around 1 nm was found in either hybrid or pure CER networks synthesized without implication of any porogen, i.e., due to intrinsic microporosity representing free volume (voids) arising from the formation of triazine-based macrocycles in a rigid network structure. The materials obtained have shown high gas adsorption capacities (H_2_, CO_2_, N_2_) with good selectivities for several gas pairs, namely CO_2_/N_2_, CO_2_/CH_4_, etc. [16–18].

Recently, we have reported on several novel approaches for producing mesoporous thin film CER samples via selective alkaline hydrolysis [19] or partial extraction [20] of reactive poly(ε-caprolactone) (PCL) sub-chains from CER/PCL hybrid networks. We also successfully created nanoporous CER-based networks via irradiation of thin films by α-particles, followed by chemical track etching [21]. Lately, we have developed a simple method for generating nanoporous film materials based on CERs by varying the rate and degree of completion of curing associated with the polycyclotrimerization of dicyanate monomer. A series of nanoporous CER films obtained by such incomplete conversion technique were thus synthesized [22]. We have also implemented a chemically induced phase separation route toward the formation of nanoporous pure CER networks by using inert high-boiling temperature porogens, e.g., dimethyl- and dibutyl phthalates, during network formation, followed by their quantitative removal [23]. Later on, we have enlarged the concentration range and the phthalate nature used for pore generation [24, 25]. Moreover, we have found relevant effects of porogen molecules trapped inside the crosslinked CER matrices after pore generation procedure on both structure and physico-chemical characteristics of the resulting porous CER-based film materials [24, 25].

Further studies on kinetics of isothermal annealing of such nanoporous CER-based films during a long-term (up to 48 h) treatment at temperatures far below their glass transition (i.e., at 50–150 °C) have shown that a steady state was reached, after 5–20% of mass loss associated with desorption processes of both moisture/water and porogen molecules trapped in the densely crosslinked CER matrices [26]. After annealing, their chemical structure and thermal performances were not significantly changed, whereas a certain effect on the main porosity features was noticed [25, 26]. However, ensuring complete removal of high-boiling temperature porogens from CER matrices still remains challenging because below their glass transition temperatures, some porogen residues could be “fully blocked” within the CER network structure. Therefore, one can assume that an additional thermal treatment has to be performed above the glass transition of the CERs formed to allow for devitrification of incompletely cured CER fragments, thus leading to the release of the trapped porogen molecules. However, in this case, the porogen evaporation might be supplemented by post-curing of dangled CER chains finally resulting in modified porosity of the more densely crosslinked resulting CER material.

Herein, the effect of short-term thermal processing at 250 °C on structural peculiarities and gas transport properties of the nanoporous CER film materials produced have therefore been analyzed in details. Gas transport properties in polymers are governed by a diffusion/solubility mechanism which is closely related to polymer chain mobility and to free volumes available for gas sorption and diffusion process. Although some gas sorption data have recently been determined for micro-and mesoporous polycyanurate networks [14, 15], it has to be noted that no data concerning both gas diffusion and sorption properties have hitherto been reported for such CER networks. It is worth noting that both parameters are important and useful to probe the structure and chain mobility of thermosets. As an example, previous studies performed on glassy epoxy networks [27] showed that increasing the network crosslink degree led to an increase in gas solubility. It was observed that the gas diffusion simultaneously dropped due to the increase in the glass transition temperature. The same research work also revealed an increase in the diffusion coefficient within the network in the presence of a plasticizer (dibutyl phthalate) associated with a decrease in the gas solubility. These results were assigned to a raise in the chain mobility and a descent of the free holes available for gas sorption [27].

In the present work concerning nanoporous CER films obtained through the use of phthalates as high-boiling temperature porogens, gas transport properties, namely diffusion coefficient, solubility coefficient, and resulting permeability coefficient, are determined for a series of gases of different kinetic diameters, and the evolution of gas transport parameters of nanoporous CERs as a function of the initial porogen content and the processing steps applied are discussed regarding the network structure.

### Materials

1,1′-bis(4-cyanatophenyl)ethane (dicyanate ester of bisphenol E, DCBE), under the trade name Primaset LECy, was kindly supplied by Lonza, Switzerland. Cobalt acetyl acetonate (Co(AcAc)_2_) and nonylphenol were used as a catalytic complex; these compounds were supplied by Sigma-Aldrich. High-boiling temperature porogens, including dibutyl phthalate (DBP, purity ≥99%, bp ~340 °C, *M* ~278 g mol^−1^, mp ≈−35 °C) and dimethyl phthalate (DMP, purity ≥99%, bp ~282 °C, *M* ~194 g mol^−1^, mp ≈0–2 °C) were supplied by Sigma-Aldrich. All the chemicals were used as received.

### Preparation of CER-Based Films

In order to ensure high porosity of the resulting films, the content of phthalates in CERs was chosen as high as possible. It is noteworthy that some limitation in the in situ formation of crosslinked CER networks was found experimentally, due to a dilution effect, and the ultimate DBP and DMP concentrations were reached at 30 and 50 wt% with respect to DCBE, respectively.

DCBE was mixed with either DBP or DMP in a given ratio, and the catalytic system comprising 100 ppm of Co(AcAc)_2_ and 2 phr of nonylphenol was added. Finally, the mixture was placed into a PTFE-coated mold and heated through the following step-by-step schedule: 150 °C for 5 h, then 180 °C for 3 h, and finally 210 °C for 1 h. The films obtained with a thickness up to 300 μm were subjected to extraction with acetone in a Soxhlet apparatus for 24 h. The following codes were applied to the nanoporous samples studied: CER_DBP30_, CER_DMP30_, and CER_DMP50_, where the subscripts indicate the type and the initial content (wt%) of the porogen used (Table 1). Non-extracted porogen residues were then removed by additional heating at 250 °C for 2 h. The resulting CER films were named as follows: a-CER_DBP30_, a-CER_DMP30_, and a-CER_DMP50_, where the prefix “a-” in sample codes distinguishes the annealed nanoporous CER films.

**Table 1:** Main porosity characteristics for nanoporous CER-based films studied after Soxhlet extraction

Sample code	Pore size distribution^a^ (nm)	Average pore diameter^a^ *D*_p(av)_ (nm)	Total pore volume^a^ *V*_p_ (cm^3^ g^−1^)
CER_DBP30_	15–160	49	0.12
CER_DMP30_	15–180	33	0.19
CER_DMP50_	20–185	34	0.34

^a^Values as determined by DSC-based thermoporometry (see “Methods”)

As a conclusion, CER/DBP (70/30), for example, is referred to the CER film containing initially 30 wt% of DBP; CER_DBP30_ is related to the corresponding film obtained after Soxhlet extraction, and a-CER_DBP30_ is used to name the sample obtained after Soxhlet extraction and additional thermal treatment at 250 °C for 2 h.

## Methods

Fourier transform infrared (FTIR) studies were performed on a Bruker model Tensor 37 spectrometer between 4000 and 800 cm^−1^. For each spectrum, 32 consecutive scans with a resolution of 0.6 cm^−1^ were averaged. The measurements were carried out in the attenuated total reflection (ATR) mode.

Differential scanning calorimetry (DSC) measurements were performed on a Perkin Elmer DSC 8500 calorimeter under nitrogen atmosphere by heating from −40 to 300 °C at a rate 20 °C min^−1^. The glass transition temperature was defined as a midpoint-by-half-height of glass transition on the corresponding DSC thermograms obtained after a second heating run. Temperature and heat flow calibrations were achieved by measuring the melting characteristics of indium.

DSC-based thermoporometry was used as an independent quantitative technique for determining pore sizes, pore size distributions, and total pore volumes (see Table 1). Basic principles and methodology of DSC-based thermoporometry technique are well known [28, 29]. In this study, thermoporometry was performed using water as a penetrating liquid. From the melting thermograms of water contained in the porous samples, Eq. (1)–(3) were applied for determination of pore diameter (*D*p), pore size distribution (*dV/dR*), and heat flow Δ*H(T)*:

1 $$ {D}_p=2\;\left(0.68-\frac{32.33}{T_m-{T}_{m0}}\right), $$

2 $$ d V/ d R=\frac{dq/ d t\;{\left({T}_m-{T}_{m0}\right)}^2}{32.33\;\rho \kern0.2em \nu\;m\kern0.1em \varDelta H(T)}, $$

3 $$ \varDelta H(T)\kern0.5em =332+11.39\;\left({T}_m-{T}_{m0}\right)+0.155\;{\left({T}_m-{T}_{m0}\right)}^2, $$

where *T*_m_ and *T*_m0_ are the melting temperatures of crystallites of confined (inside pores) and bulk water, respectively; *dq/dt, ρ, v, m*, and Δ*H(T)* are the heat flow recovered by DSC, the water density, the heating rate, the sample mass, and the melting enthalpy of water, correspondingly. Total pore volume (*V*_p_) was determined as a result of integration of *dV/dR = f* (*D*p).

Scanning electron microscopy (SEM) analyses were performed on a MERLIN microscope from Zeiss equipped with Inlens and SE2 detectors using an accelerating voltage of 4 kV. Prior to analyses, the films were cryofractured and coated with a Pd/Au alloy (4 nm thickness) in a Cressington 208 HR sputter-coater.

Thermogravimetric analysis (TGA) measurements were performed in a Thermo Scientific Heraeus® programmable drying oven in isothermal mode at 250 °C under air atmosphere for 24 h. To determine the mass evolution as a function of time, the samples were periodically removed from the oven and weighed.

Permeation measurements were performed at 20 °C for helium (He), oxygen (O_2_), and carbon dioxide (CO_2_) with respective kinetic diameters of 2.65, 2.92, and 3.23 Ǻ, respectively [30]. The CER-based samples with a useful area of 3 cm^2^ and a constant thickness around 150 μm were placed between the upstream and downstream compartments of the permeation cell. A secondary vacuum desorption step was performed prior to each permeation experiment. The permeation measurements were carried out under an upstream pressure, *P*_1_, equal to 3 bars. The downstream pressure, *P*_2_, was estimated as a function of time. The permeability coefficient, *P*, was calculated from the slope of the linear time dependence of *P*_2_ in a steady state, and the diffusion coefficient, *D*, was deduced from the time lag, *θ*, as determined by the extrapolation of the steady-state line on the time axis:

4 $$ D=\frac{e^2}{6\theta}, $$

where *e* is the film thickness. *D* was expressed in cm^2^ s^−1^ and *P* in barrer.

Assuming a Fickian transport mechanism, the solubility coefficient, *S*, was calculated from the following equation [30]:

5 $$ P= D\cdot S, $$

where *S* was expressed in cm^3^_STP_ cm^−3^ cm_Hg_^−1^.

The sorbed CO_2_ concentration (*C*_CO2_) was calculated from the solubility parameter by taking into account the gas pressure *P*_1_ at which the permeation experiments were performed.

6 $$ {C}_{\mathrm{CO}2}= S\cdot {P}_1. $$

The accuracy on the transport coefficients was estimated to be equal to ±8%.

The ideal selectivity for a gas pair (*α*_*A*/*B*_) was also calculated by the ratio of the gas permeability coefficients as follows:

7 $$ {\alpha}_{A/ B}=\frac{P_A}{P_B}. $$

## Results and Discussion

### Chemical Structure and Morphology of CER-Based Films

**Fig. 1:**
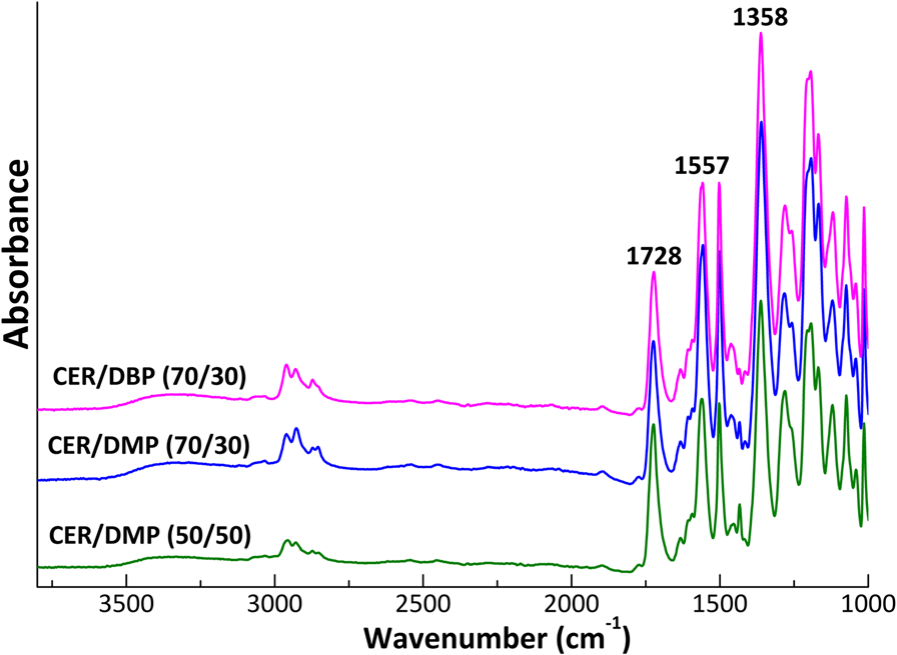
FTIR spectra of CER/phthalate precursory networks (i.e., before porogen extraction)

FTIR analysis of chemical structures associated with the cured CER networks containing 30–50 wt% of the phthalates with different alkyl chains confirmed total transformation of the reactive –O–C≡N groups from DCBE with stretching vibrations around 2280–2220 cm^−1^ into –C=N– and phenylene–oxygen–carbon groups of triazine rings with stretching absorption bands at 1358 and 1557 cm^−1^, respectively (Fig. 1). Dimethyl- and dibutyl phthalate components in the corresponding CER/phthalate samples displayed a strong carbonyl absorption band with maximum around 1728 cm^−1^.

**Fig. 2:**
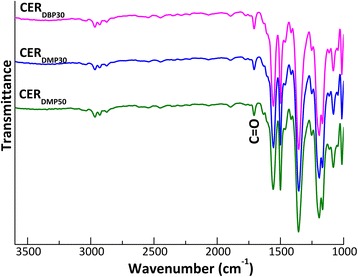
FTIR spectra (transmittance mode) of nanoporous CERs (i.e., after porogen extraction)

As previously disclosed [23–25], after acetone extraction of phthalates, the C=O band disappeared, thus evidencing their complete removal from the CER networks synthesized. Analysis of the extracted CER-based samples by FTIR measurements in transmittance mode allowed scanning of bulk material and revealed more pronounced C=O bands, thus giving evidence of the occurrence of a certain amount of phthalates entrapped within the corresponding CER crosslinked structures after extraction (Fig. 2).

**Fig. 3:**
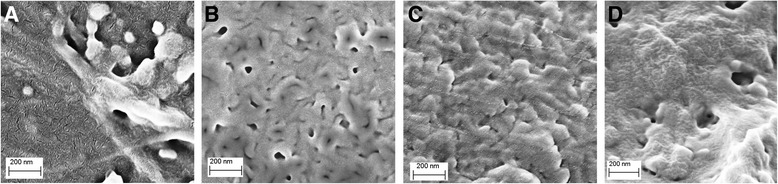
Typical SEM micrographs of nanoporous CER_DMP30_ (**a**, **b**), CER_DMP50_ (**c**, **d**) films before (**a**, **c**) and after (**b**, **d**) annealing at 250 °C for 24 h

Our previous investigation of long-term isothermal annealing at 250 °C of nanoporous CER film materials after porogen extraction showed a continuous drop in weight of the samples investigated. More specifically, annealing at 250 °C for 24 h led to irreversible changes in chemical structure of the synthesized nanoporous CER networks [25, 26]. A comparative analysis of SEM micrographs (Fig. 3) for the nanoporous samples before and after 24 h annealing at 250 °C revealed the generation of well-defined nanoporous structure in the CER-based films obtained after extraction of the phthalates with pore sizes ranging from *Dp* ~10 to 120 nm. It is noteworthy that after annealing at 250 °C for 24 h, a lower pore density within nanoporous structures could be observed (Fig. 3b, d).

**Fig. 4:**
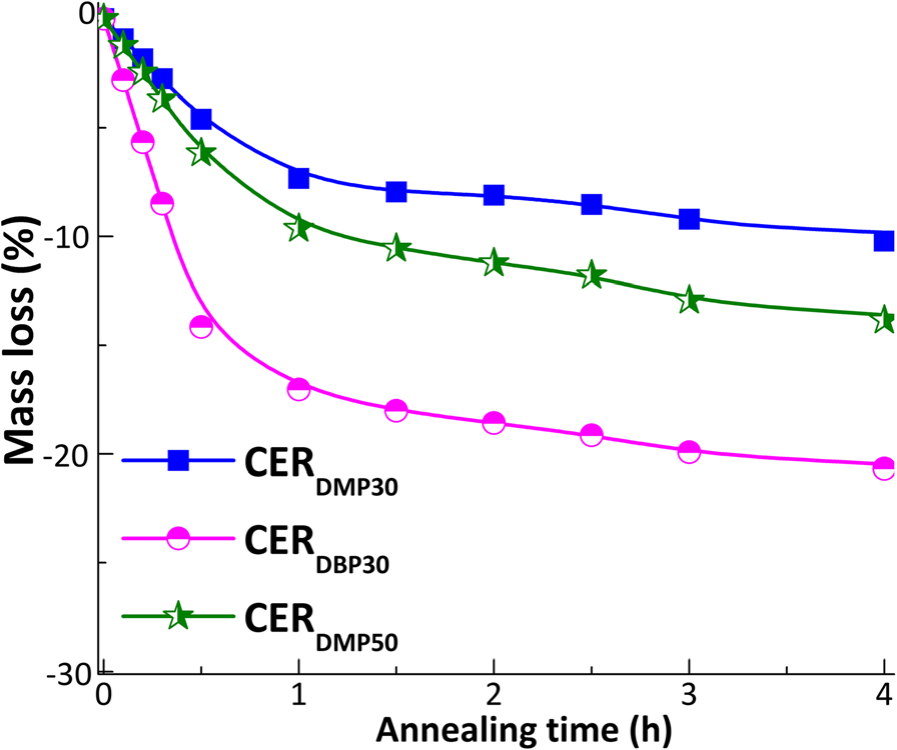
Isothermal thermogravimetric curves obtained for nanoporous CER samples annealed at 250 °C

A more detailed analysis of the sample mass loss, while annealing at 250 °C (Fig. 4), revealed an inhibition period for the mass loss process in the early annealing stages (after 2–4 h) [26]. Depending on the type and amount of the porogen used, the most rapid mass loss (∆*m*) was observed after ~1 h of annealing (∆*m* = 7.4–17%), whereas additional annealing for 3 h only caused an additional ∆*m* drop by 2.9–4.2%, overall reflecting both moisture and porogen evaporation [26].

**Fig. 5:**
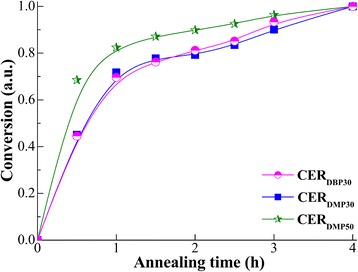
Dependence of conversion on annealing duration at 250 °C for CER-based nanoporous samples

In order to get a better insight into one such mass loss process, the isothermal curves were replotted in terms of conversion as a function of annealing time, where “zero-point” represents the initial weight of the nanoporous CER film investigated and 100% conversion stands for the mass value after 4 h of annealing at 250 °C (Fig. 5).

**Fig. 6:**
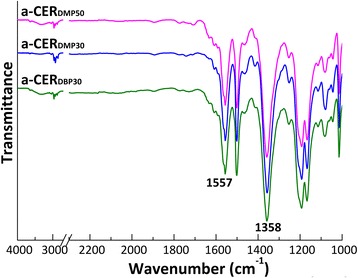
FTIR spectra of nanoporous CERs after extraction and additional heating at 250 °C for 2 h

One could see that relatively constant weight loss plateau (a quasi-steady state) for all the samples investigated could be reached after short-term annealing. FTIR analysis of the nanoporous CER samples annealed for 2 h (Fig. 6) showed similar chemical structures as compared to those for non-annealed analogues. At that, the former spectra revealed the absence of either residual porogen or any degradation products.

Consequently, obtaining phthalate-free nanoporous CER materials by the chemically induced phase separation approach using both DMP and DBP required applying additional thermal post-treatment [25]. However, sample annealing at elevated temperatures (i.e., above the glass transition) may also affect the chain mobility as well as and the gas transport properties.

### Gas Transport Properties

The values of the gas transport parameters measured for the different CER films are reported in Table 2. It was impossible to determine with a good accuracy the diffusion coefficient values for helium because the low kinetic diameter of this molecule made its diffusion rate fast, leading to very low time lag values caused by the thickness of the films investigated.

**Table 2:** Gas transport parameters for CER films under investigation

Sample code	*P*_He_ (barrer)	*P*_O2_ (barrer)	*D*_O2_ 10^−8^ (cm^2^ s^−1^)	*S*_O2_ 10^−2^ (cm^3^_STP_ cm^−3^ cm_Hg_^−1^)	*P*_CO2_ (barrer)	*D*_CO2_ 10^−8^, (cm^2^ s^−1^)	*S*_CO2_ 10^−2^, (cm^3^_STP_ cm^−3^ cm_Hg_^−1^)	*C*_CO2_ (cm^3^_STP_ cm^−3^)	*α*_He/O2_	*α*_He/CO2_
CER/DBP (70/30)	4.49	0.61	4.55	0.13	3.69	1.56	2.40	5.62	7.4	1.2
CER/DMP (70/30)	3.14	0.37	2.00	0.18	1.40	0.52	2.70	6.07	8.5	2.2
CER/DMP (50/50)	4.13	0.56	4.66	0.12	2.87	1.21	2.37	5.33	7.4	1.4
CER_DBP30_	6.82	0.61	0.58	1.05	3.15	0.22	14.20	32.17	11.2	2.2
a-CER_DBP30_	7.30	0.45	0.64	0.70	1.46	0.16	9.30	20.92	16.2	5.0
a-CER_DMP30_	7.30	0.72	0.94	0.76	2.28	0.22	10.10	22.81	10.1	3.2
a-CER_DMP50_	7.00	1.08	1.10	0.98	3.13	0.26	12.10	27.22	6.5	2.2

Different trends could be observed from the transport parameter values reported in Table 2. For all the membranes studied, the permeability coefficient values increased going from O_2_ through CO_2_ to He. These variations were in agreement with the general trends observed for polymers. The high permeability level obtained for helium could be assigned to its low kinetic diameter and fast diffusion rate. Oxygen exhibited the lower permeability values due to the combination of its low diffusion rate and low solubility. The intermediate values of permeability obtained for CO_2_ could be assigned to the high solubility of this molecule. The permeability values of the developed membranes were similar to those measured in the same conditions on polyetherimide [31]. They were significantly higher than those obtained on polyamides [32, 33] or on a commonly used polyimide (based on 3,3′,4,4′-benzophenone tetracarboxylic dianhydride and 4,4′-diaminodiphenylether) [34].

In the film series investigated, one could observe that the lowest gas permeability and solubility coefficient values and the highest gas diffusion rates were obtained before the Soxhlet extraction step. The phthalate molecules were located in the network free volumes; therefore, the latter were not available for gas sorption. It could also be noticed that increasing the DMP content in the CER network had a much higher impact on the diffusion coefficient than on the solubility coefficient. In the non-extracted network series, the permeability seemed then to be governed by the kinetic parameter of the transport.

The high value of the diffusion rate could be assigned to significant network chain mobility due to the plasticizing effect induced by the phthalate moieties. Indeed, the non-extracted networks exhibited low glass transition temperature values with respect to the extracted ones (Table 3). After extraction, a narrowing of the Δ*T*_g_ range and a shift of the glass transition temperature to higher values were observed for all the samples studied. Discussion on the interpretation of the Δ*T*_g_ values presented by Saiter et al. [35] has shown that the higher the Δ*T*_g_ values, the larger the width of time distributions characterizing molecular dynamics, and therefore the higher long-scale structural disorder in the materials is expected.

**Table 3:** DSC data for CER-based samples under investigation

Sample code	Glass transition temperature, *T*_g_ (°C)			
	Onset	Midpoint	End	Δ*T*_g_^a^
CER/DBP (70/30)	80	104	129	49
CER/DMP (70/30)	74	96	119	45
CER/DMP (50/50)	88	105	123	35
CER_DBP30_	214	225	236	22
CER_DMP30_	210	224	238	20
CER_DMP50_	204	214	224	28

^a^The glass transition temperature range (Δ*T*_g_) was calculated as the difference between end and onset temperature values associated with the glass transition zone

The impact of the extraction step was investigated on CER/DBP (70/30) sample (see Table 2). Indeed, previous analysis performed on CER films based on higher porogen contents evidenced the presence of a certain degree of heterogeneity after Soxhlet extraction [25]. This could make difficult the interpretation of the gas transport behavior. The data presented in Table 2 revealed that the extraction performed on CER/DBP film had a significant effect on He permeability, whereas it only slightly modified O_2_ and CO_2_ permeability values. The low variation of the oxygen and carbon dioxide permeability values observed for this network could be explained by the opposite variation of the diffusion and solubility coefficients. The extraction step led to the formation of nanometer-sized pores, thus explaining the significant increase observed for gas solubility. However, the loss of DMP simultaneously led to a significant decrease of the chain mobility, thus making the diffusion rate slower. The significant impact of the extraction step observed on He permeability could be assigned to the small kinetic diameter of this molecule and its lower sensitivity to a variation of polymer chain segmental mobility. The expected decrease of the diffusion coefficient due to chain mobility restriction should then be much less important for this gas in comparison to gases constituted of larger molecules. As a result, a significant increase of helium permeability was observed for the films after extraction causing an increase of the He/CO_2_ and He/O_2_ selectivity values by a factor ~1.5–1.8 with respect to the non-extracted analogues. Last, it is noteworthy that the amount of CO_2_ sorbed in the CER_DBP30_ film (e.g., *C*_CO2_ ~32 cm^3^_STP_ cm^−3^, see Table 2) was similar to those reported on micro-and mesoporous polycyanurate networks, despite different pore contents and sizes. Indeed, the films prepared by Yu et al. [14, 15] were characterized by smaller pore size dimensions but higher total pore volumes as compared to those investigated in the present work.

The effect of an additional heating of the nanoporous films above their glass transition temperatures (i.e., at 250 °C) was also estimated. Comparative studies of transport properties of CER_DBP30_ membranes before and after annealing showed that one such post-treatment did not significantly modify He permeability, while it led to a decrease in CO_2_ and O_2_ permeability coefficient values. By taking into account the uncertainty, one could also conclude that O_2_ and CO_2_ diffusion coefficients did not vary, whereas the gas solubility diminished after the annealing step. It was shown that during annealing at 250 °C, porogen evaporation occurred and post-curing of dangled CER chains might also take place. As the CER crosslink degree was already high before the annealing step, it could be assumed that the variation of *T*_g_ value induced by the additional thermal post-curing was not high enough to have a significant impact on *D* values. However, as the annealing at 250 °C took place in the rubbery state, changes of porosity occurred due to possible chain reorganization within the CER networks. The decrease of the gas solubility was indicated thus a decrease of the global porosity volume and the increase of gas selectivity could be related to a decrease of the mean pore sizes.

The CO_2_ and O_2_ transport parameters measured for a-CER_DMP30_ sample were systematically slightly higher than those determined for a-CER_DBP30_. The lower molecular weight of DMP as compared to that of DBP as well as lower affinity of DBP to CER monomer (DCBE) [23, 24] led to introducing a higher amount of porogen molecules in CER/DMP samples than in CER/DBP systems. After porogen desorption, these facts resulted in higher pore volume contents for CER/DMP samples as compared to CER/DBP ones. This effect was emphasized by increasing the DMP amount from 30 to 50 wt%. A certain growth of pore volume had a higher impact on the behavior of CO_2_ and O_2_ in comparison to He, thus showing again the importance of the diffusant kinetic diameter on the transport mechanism in this nanoporous thermosetting polymer series.

## Conclusions

Nanoporous membranes based on thermostable densely crosslinked CER networks were generated by using the chemically induced phase separation approach via the polycyclotrimerization of DCBE in the presence of 30–50 wt% of inert high-boiling temperature phthalates as porogens, followed by their extraction from the CER networks and subsequent short-term annealing at 250 °C. The nanoporous structure for all the CER-based films developed was characterized by using SEM and DSC-based thermoporometry techniques. The average pore diameters were varied from ~15 to 50 nm, and pore volume values were found in the range of 0.08–0.30 cm^3^ g^−1^. It is noteworthy that increasing phthalate contents led to larger pore diameters and higher pore volumes.

Interestingly, the nanoporous thermosetting materials demonstrated a significant increase in He permeability after porogen extraction. Moreover, all membranes exhibited higher CO_2_ and O_2_ sorption abilities and increased He/O_2_ and He/CO_2_ selectivity factors after porogen desorption (i.e., after annealing step). It was shown that both chain mobility and free volumes governed the gas transport properties before porogen extraction, whereas the pore volume content and pore size became predominant factors in the case of the CER membranes obtained. Additional annealing of these high *T*_g_ systems in rubbery state clearly led to a modification of the porous network structure, which allowed for an increase in the separation ability between low and higher size gas molecules in glassy state.

Nanoporous thermosetting membranes possessing high thermal stability are expected to be promising materials for advanced applications in diverse areas, including filtration, separation, and purification techniques.

## Abbreviations

CER: Cyanate ester resin
DBP: Dimethyl phthalate
DCBE: Dicyanate ester of bisphenol E
DMP: Dibutyl phthalate
